# Nontuberculous Mycobacteria from Household Plumbing of Patients with Nontuberculous Mycobacteria Disease

**DOI:** 10.3201/eid1703.101510

**Published:** 2011-03

**Authors:** Joseph O. Falkinham

**Affiliations:** Author affiliation: Virginia Polytechnic Institute and State University, Blacksburg, Virginia, USA

**Keywords:** Bacteria, nontuberculous mycobacteria, tuberculosis and other mycobacteria, household water, plumbing, biofilms, DNA fingerprinting, research

## Abstract

To determine whether plumbing could be a source of nontuberculous mycobacteria (NTM) infection, during 2007–2009 I isolated NTM from samples from household water systems of NTM patients. Samples from 22/37 (59%) households and 109/394 (28%) total samples yielded NTM. Seventeen (46%) of the 37 households yielded >1 *Mycobacterium* spp. isolate of the same species as that found in the patient; in 7 of those households, the patient isolate and 1 plumbing isolate exhibited the same repetitive sequence-based PCR DNA fingerprint. Households with water heater temperatures <125°C (<50°C) were significantly more likely to harbor NTM compared with households with hot water temperatures >130°F (>55°C) (p = 0.0107). Although households with water from public or private water systems serving multiple households were more likely to have NTM (19/27, 70%) compared with households with a well providing water to only 1 household (5/12, 42%), that difference was not significant (p = 0.1532).

Nontuberculous mycobacteria (NTM) are opportunistic pathogens found in the environment (e.g., water and soil) and cause life-threatening infections in humans, other mammals, and birds (*1**,**2*). The incidence of NTM disease in Canada and the United States seems to be increasing (*3**–**5*). In Toronto, Ontario, Canada, NTM disease incidence rose from 1.5 to 9.0 cases per 100,000 population during 1997–2003 (*3*). The most common NTM infecting persons in the United States are *Mycobacterium avium,* *M. intracellulare*, and *M. avium* complex (MAC) (*6*). Infections occur in immunodeficient (e.g., HIV/AIDS) and immunosuppressed (e.g., cancer and transplant) patients and nonimmunosuppressed persons with the classic risk factors for mycobacteria infection, which include exposure to dust or smoke and underlying lung disease (*6**,**7*). Cystic fibrosis (*8*), heterozygosity for mutations in the cystic fibrosis transmembrane conductance regulator gene (*9*), and α-1-antitrypsin deficiency (*10*) predispose persons to NTM disease. Elderly, slender women lacking any of the classic risk factors for NTM disease are also at risk for NTM pulmonary disease (*11**–**13*). The major manifestation of NTM infection in the immunocompetent host is pulmonary disease, whereas disseminated disease (i.e., bacteremia) is found in patients with AIDS and other immunosuppressed persons (*6*).

NTM, particularly *M. avium* and *M. intracellulare*, have been recovered from a variety of environmental niches with which humans come in contact, especially drinking water (*14**–**19*). NTM are not transient contaminants of drinking water distribution systems; rather, the NTM grow and persist in plumbing (*19**,**20*). For example, numbers of mycobacteria increase in pipes as the distance from the treatment plant increases (*19*). NTM cell surface hydrophobicity results in disinfectant resistance and a predilection to attach to surfaces where NTM grow and form biofilms (*21**,**22*) that further increase disinfectant resistance (*23*). Because disinfectants inhibit the competing microflora, the slow-growing NTM can grow on the available nutrients in the absence of competition. *M. avium* can grow in drinking water at concentrations of assimilable organic carbon of >50 µg/L (*24*). Thus, there is strong reason to hypothesize that NTM can colonize and persist in household plumbing.

Sources of human infection with NTM, including MAC, have been found in water (*18*) and potting soil (*25*). Notably, *M. avium* was detected in water aboard the Russian space station Mir (*26*). Recently, researchers found that the DNA fingerprints of several *M. avium* isolates recovered from the shower of an *M. avium*–infected patient were almost identical to isolates recovered from the patient, indicating that the household water could have been the source of the patient’s pulmonary disease (*27*). Despite that evidence, several publications have documented low frequency of recovery of MAC from household water samples (*17**,**28**–**30*). Such low recovery rates of *M. avium* and *M. intracellulare* could be because water samples, not biofilm, were collected. As MAC preferentially attaches to surfaces (*21**–**23*), MAC may be at low numbers in water samples. Furthermore, in the studies cited above, a low number (<4) of samples were collected from individual households. Recovery of multiple NTM or MAC isolates is necessary because of the clonal variation of MAC (*25**,**27*). The pilot study described here isolated, enumerated, and DNA fingerprinted NTM from households of patients with NTM to test the hypothesis that household plumbing could be a source of their NTM infection.

## Methods

### Patients and NTM Isolates

NTM patients were recruited to participate in studies of their household water systems through the auspices of Nontuberculous Mycobacteria Research and Information, Inc. Informed consent was obtained from each participating patient, and the study was reviewed by the Virginia Tech Institutional Research Board and granted exempt status. NTM isolates from the patients, if possible, were obtained through collaborating physicians and mycobacteriology laboratories. In some instances multiple patient isolates of different species were found. A questionnaire was provided to each patient to obtain information about the household plumbing.

### Household Water and Biofilm Samples

Sterile containers and swabs were sent to each collaborating patient household. Directions for collection of hot and cold water samples (500 mL) and biofilms/sediment from water taps and showerheads by using swabs were provided. If the patient thought that infection might have occurred as a result of exposure to soil, soil samples were collected. In some cases filters (fiber, activated charcoal, and reverse osmosis) were collected. All samples were returned at ambient temperatures by express courier service to the Mycobacteriology Laboratory in the Department of Biological Sciences at Virginia Tech.

### Isolation and Identification of Mycobacteria

Mycobacteria in water and swab (taps and filters) samples were counted and isolated as described (*27*). Soil samples were processed as described (*25*). Most acid-fast colonies picked for identification and enumeration were small (1-mm diameter after 14 days at 37°C), unpigmented to yellow, and resembled either the transparent or opaque types previously reported (*17*). Acid-fast isolates were identified by nested PCR of the 16S rRNA gene (*31*) and PCR amplification and analysis of restriction endonuclease digestion fragments of the heat-shock protein 65 (*hsp65*) gene (*32*).

### Fingerprinting Patient and Environmental Isolates

In those instances in which the *Mycobacterium* species from the patient and household water system isolates were the same, isolates were fingerprinted by repetitive sequence-based PCR (*rep*-PCR) (*33*). Matches were confirmed by use of GelCompar II software (Applied Maths, Inc., Austin, TX, USA).

## Results

### Household Plumbing Samples

Samples for NTM isolation were received from 31 collaborating patients throughout the United States and Canada: Arizona, California, Colorado, Connecticut, Florida, Georgia, Michigan, New Jersey, New York, Pennsylvania, Texas, Vermont, Virginia, and Wisconsin, USA; and Ontario, Canada. Six patients each had 2 residences and sent samples from each residence.

### NTM Isolation

The isolates from the 31 patients with NTM infection included *M. avium* (9), *M. intracellulare* (6), MAC (11), *M. abscessus* (4), and *M. xenopi* (1). Isolates could not be obtained from 11 patients, thus preventing *rep*-PCR fingerprinting even in those instances where household isolates belonged to the same species. Thus, the total number of patient isolates available for fingerprinting was only 20. All putative *Mycobacterium* spp. isolates recovered from samples were identified, and 45% of NTM-positive households (10/22) and 1.5% of NTM-positive samples (6/394) yielded >1 NTM species (Table 1). The average number of different NTM species per household was 1.9 (range 1–5 NTM species/household). In those instances where the *Mycobacterium* species of the patient and their household plumbing isolates were the same (e.g., *M. avium*), all isolates belonging to the same species as the patient were subject to *rep*-PCR fingerprinting. Household isolates included *M. avium* (10), *M. intracellulare* (10), *M. malmoense* (5), *M. szulgai* (3), *M. chelonae* (2), *M. gordonae* (6), and 1 each of *M. scrofulaceum*, *M. terrae*, and *M. trivale* (Table 1). Samples were coded with the first 2 or 3 letters representing each patient, a letter representing sample type (W, water; Sw, swab [biofilm]; S, soil), a number for sample number from a household collection, and the final number for the isolate from the sample; thus, ML-W-6-2 is the second water isolate from the sixth sample collected from patient ML’s household.

**Table 1:** Characteristics of NTM isolated from samples from household plumbing of patients with NTM infection, 2007–2009*

Characteristic	Value
No. patients	31
No. households sampled	37†
Households with NTM	22/37 (59)
Households with >1 NTM species	10/22 (45)
Total no. samples collected	394
Samples with NTM	109/394 (28)
Samples with >1 NTM species	6/394 (1.5)
Households with NTM of same species as patient	17/37 (46)
Household and patient NTM share same fingerprint	7/17 (41)

* Values are no. positive results/no. samples in category (%) except as indicated. NTM, nontuberculous mycobacteria. †Six patients had 2 residences and submitted samples from each.

NTM were isolated from water, biofilm, filter, or soil samples from 22 (59%) households sampled and from 109 (28%) of 394 samples. There was a positive correlation between the number of samples collected per household and the number of NTM-positive samples (r = 0.4581). In 8 households >50% of the samples yielded NTM, and in 7 households no NTM were isolated. Seventeen of the 37 household sample collections had at least 1 sample that yielded an NTM isolate that belonged to the same species as that of the patient. Among those 17 households, at least 1 NTM isolate from 7 households exhibited the same *rep*-PCR fingerprint as that of the patient. Specifically, the Figure illustrates matching *rep*-PCR band patterns of patient isolate ML-P-1 (lane 3) and shower water isolate ML-W-6-2 (lane 4) from the patient’s home and patient isolate TC-P-1 (lane 10) and tap water isolate TC-W-2-2 (lane 12) from the patient’s home. Matches were confirmed by use of GelCompar II software (Applied Maths, Inc.). Furthermore, the Figure also illustrates the relative similarity in *rep*-PCR band patterns of patients and their household isolates and the wide differences between isolates of different patients (compare lanes 3–4, lanes 7–8, and lanes 10–12). On the basis of diversity of band patterns and the number of bands (7–14 bands), the results confirm the discriminatory power of *rep*-PCR fingerprinting (*32*). The percentage of fingerprint matches may be an underestimate because patient isolates could not be obtained for 11/31 patients, all of whom had MAC infections.

**Figure:**
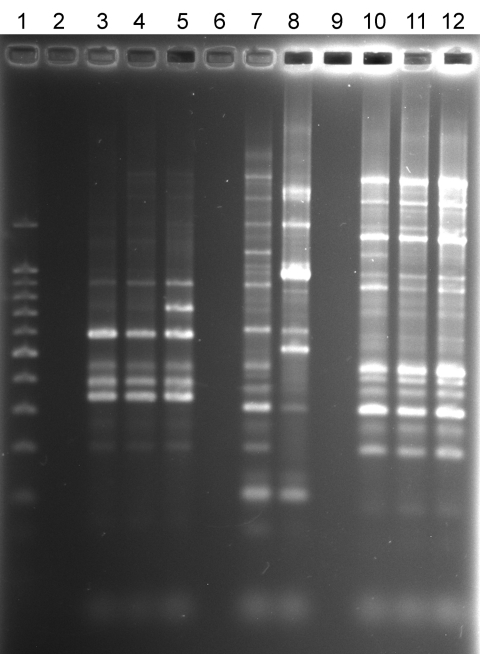
Repetitive sequence-based PCR fingerprint patterns of nontuberculous mycobacteria isolates from patients and household plumbing. Lane 1, 100-bp ladder; lane 2, no DNA control; lane 3, patient *Mycobacterium avium* isolate ML-P-1; lane 4, patient ML household *M. avium* shower water isolate ML-W-6–2; lane 5, patient ML household *M. avium* bathtub tap water isolate ML-W-8–3; lane 6, no sample; lane 7, patient *M. avium* isolate SC-P-3; lane 8, SC patient household *M. avium* water isolate SC-W-1-1; lane 9, no sample; lane 10, patient *M. avium* isolate TC-P-1; lane 11, TC household *M. avium* humidifier water isolate TC-W-4–1; lane 12, TC household *M. avium* bathroom tap water isolate, TC-W-2–2.

The frequency of NTM recovery from water (47/195, 24%), biofilm (46/165, 28%), filters (4/12, 33%), and soil samples (3/17, 18%) did not differ markedly. The highest numbers of NTM, as CFUs, were recovered from biofilms (10,371 CFU/cm^2^), with lower numbers from filters (1,987 CFU/cm^2^), soils (1,500 CFU/g), and water (157 CFU/mL). Most biofilm samples were collected by swabbing either the inside of a water tap or showerhead with a sterile swab that was immediately placed in 2 mL sterile tap water. Because the samples were shipped immediately after collection, there was little opportunity for the NTM numbers to change.

### Household Plumbing Characteristics as Determinants of NTM Presence

Review of the responses to the NTM patient questionnaire led to identification of 2 factors that seemed to influence NTM in household samples. Households with water heater temperatures <125°C (50°C) were more likely to yield NTM (17/20, 85%) compared with households in which water temperature was >130°F (55°C) (6/15, 40%) (Table 2). That difference was significant (p = 0.0107; relative risk 2.125, by Fisher exact test). Although households with water from a public or private water system were more likely to have NTM (19/27, 70%) compared with households with water from a well (5/12, 42%) that difference was not significant (p = 0.1532; relative risk 1.689 by Fisher exact test) (Table 3).

**Table 2:** Influence of water heater temperature on presence of NTM in samples from household plumbing of patients with NTM infection, 2007–2009*

Characteristic	No. households		
	NTM positive	NTM negative	Total
Water heater temperature			
<125°F (<50°C)	17	3	20
>130°F (>55°C)	6	9	15
Total no. households	23	12	35

*NTM, nontuberculous mycobacteria.

**Table 3:** Influence of water source on presence of NTM in samples from household plumbing of patients with NTM infection, 2007–2009*

Characteristic	No. households		
	NTM positive	NTM negative	Total
Water source			
Public or private piped	19	8	27
Well	5	7	12
Total no. households	24	15	39

*Two households received water from a piped system and a well. NTM, nontuberculous mycobacteria.

## Discussion

The data document the relevance of household water as a source of NTM infection. Seven (41%) of the 17 patients from whom isolates were obtained were infected with an NTM strain having the same DNA fingerprint as at least 1 NTM isolate from their household plumbing. Several characteristics of household water and plumbing are conducive to NTM survival and growth. Specifically, residual disinfectant selects for disinfectant-resistant NTM (*23*), pipe surfaces offer opportunities for biofilm formation (*21**–**23*), and low organic matter content permits growth of the oligotrophic NTM (*22**,**24*).

The frequency of samples yielding NTM (28%) reported is almost identical to the frequency of *Mycobacterium* spp. 16S rRNA sequences in biofilm (swab) samples collected from showers across the United States (*34*). In as much as that culture-independent study (*34*) did not collect samples specifically from households of NTM patients, apparently NTM are quite frequent in household water and plumbing across the United States and Canada and are not unique to household plumbing of NTM patients. In addition to exposure, host factors (*6**–**10*) are influential factors in the acquisition of NTM disease. For the study reported here, NTM patient contamination of samples was unlikely because the patients were either free of NTM in sputum or were continuing antimycobacteria therapy; none were persistently sputum positive. The low frequency of recovery of NTM by other studies (*17**,**28**–**30*) was likely because a low number of samples were collected from households. As shown here, only 28% of household samples yielded NTM, and there was a positive correlation between the number of samples collected and the recovery of NTM from household samples.

In addition to documenting the presence of NTM in households across the United States, the data from this pilot study with its relatively small sample size suggest that water heater temperature and water source could be factors influencing NTM presence. NTM were less frequently recovered from household samples whose water heater temperature was >130°C (>55°C). The relative risk of NTM presence was 2.125 for households whose water heater temperature was <125°C (<50°C). In fact, 6 of the 7 households whose patient and plumbing isolates shared identical *rep*-PCR patterns had water heater temperatures <125°C (<50°C). That association correlates with the temperature sensitivity of NTM species. For example, the time required to kill 90% of *M. avium* cells is 1,000 min at 50°C but only 54 min at 55°C; similar times were measured for *M. intracellulare* (*35*). High water heater temperatures have been associated with low numbers of *Legionella* spp. in household and other building plumbing (*36**–**39*).

It would follow that persons infected or at risk for NTM disease, e.g., slender elderly persons or cystic fibrosis transmembrane conductance regulator gene heterozygotes (*8*–*13*), consider increasing water heater temperatures. Households whose water came from a public or private water system were more likely to have NTM in household water than those whose water source was a well (p = 0.1532, relative risk = 1.689). Although not significant, that result is consistent with the fact that NTM are seldom detected in groundwater (*40*). This pilot study will be followed by an investigation to assess the influence of a variety of household plumbing characteristics in households of additional NTM patients and their neighbors.
